# Structural, composition, optical, electrical, and electrochemical characterization of three-dimensional graphene foam

**DOI:** 10.1039/d6na00510a

**Published:** 2026-08-28

**Authors:** Emma Keel, Michal Mazur, Jarosław Domaradzki, Damian Wojcieszak, Lewis Fleming, Qaisar Abbas, Michelle Ntola, Marco Caffio, Carlos García Nuñez, Des Gibson

**Affiliations:** a Institute of Thin Films, Sensors and Imaging, University of the West of Scotland Paisley Scotland UK des.gibson@uws.ac.uk; b Faculty of Microsystem Electronics and Photonics, Wroclaw University of Technology Wrocław Poland; c iGii Ltd Stirling Scotland UK; d Microelectronics Lab, James Watt School of Engineering, University of Glasgow Scotland UK

## Abstract

A comprehensive study is reported on two types of 3D graphene foam (GF) exhibiting distinct defect density, structural and functional properties. Wettability characterization demonstrates that high defect GF and low defect GF are hydrophilic, with a contact angle (CA) of approximately 14° and 72°, respectively. Optical characterization demonstrates close to zero reflectance and transmittance over a spectral range of 350 nm to 25 µm. X-ray photoelectron spectroscopy (XPS) and energy dispersive X-ray spectroscopy (EDS) provide insight into chemical composition, defect density, and functionalization, while atomic force microscopy (AFM) shows that the high defect GF (HDGF) has a pronounced surface roughness, with a maximum surface roughness of over 18 nm. However the low defect GF (LDGF) exhibits a significantly smoother and more uniform surface with a maximum surface height of 4.5 nm. Electrochemical characterization demonstrates the impact of network connectivity on charge transport, highlighting differences in resistance and percolation thresholds between the two HDGF and LDGF foam types with areal capacitances of 38 and 32 µF cm^−2^, respectively, at a current density of 0.1 mA cm^−2^. The values show 90% and 94% retention of the initial capacitance when current density was increased 50-fold. A detailed structure–property–function relationship is established for the GF materials, useful for various applications.

## Abstract 2

This paper outlines the structural, compositional, optical, electrical, and electrochemical characterization of three-dimensional graphene foam deposited using an iGii Ltd patented process.

## Graphical abstract

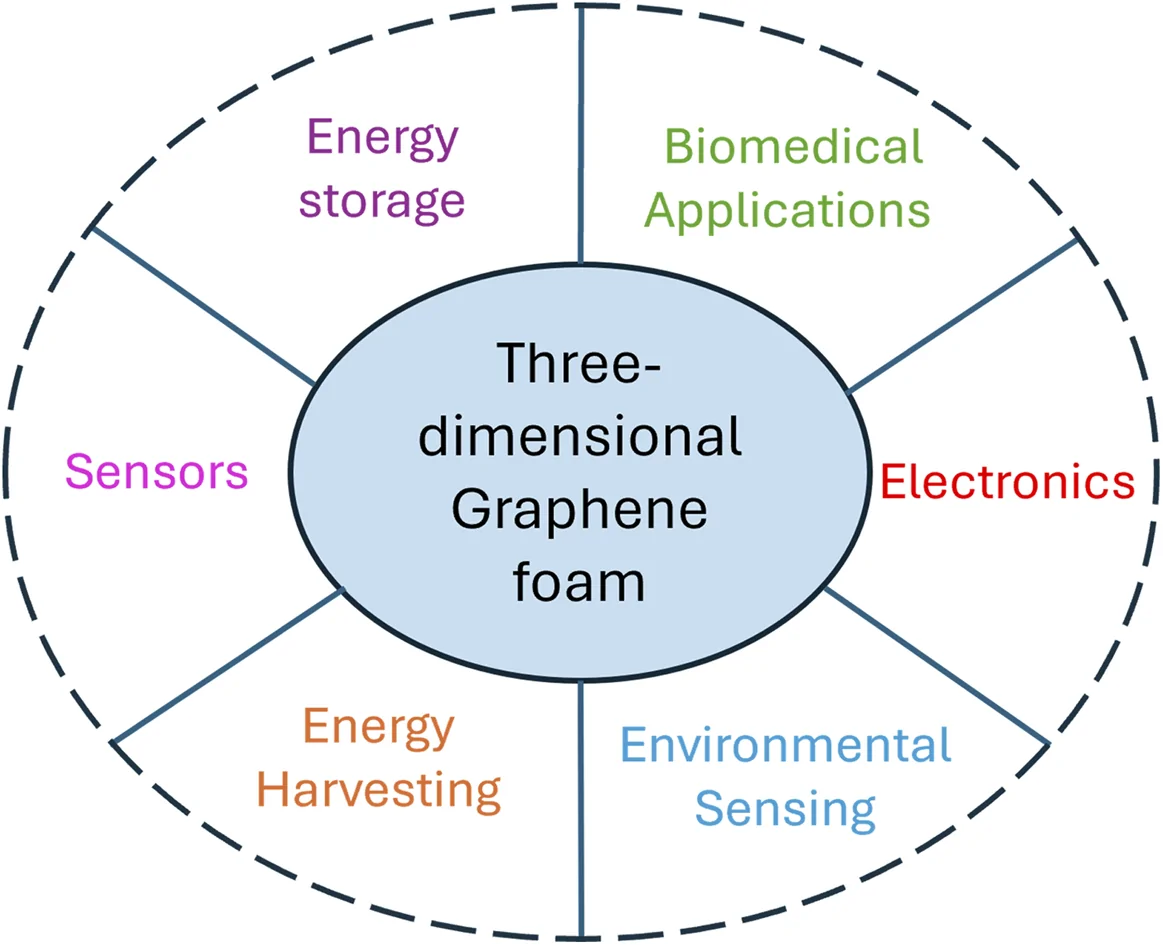

## 1. Introduction

Three-dimensional (3D) graphene foam is a unique carbon-based material that combines the remarkable intrinsic properties of graphene—such as high electrical conductivity,^1^ exceptional mechanical strength,^2^ and large theoretical surface area^3^—with the resulting properties of such a microporous, interconnected framework described in Fig. 1.^1,2,4^ Graphene foam utilized in this work was deposited using patented technology^5–8^ from iGii Ltd.^9^

**Fig. 1:**
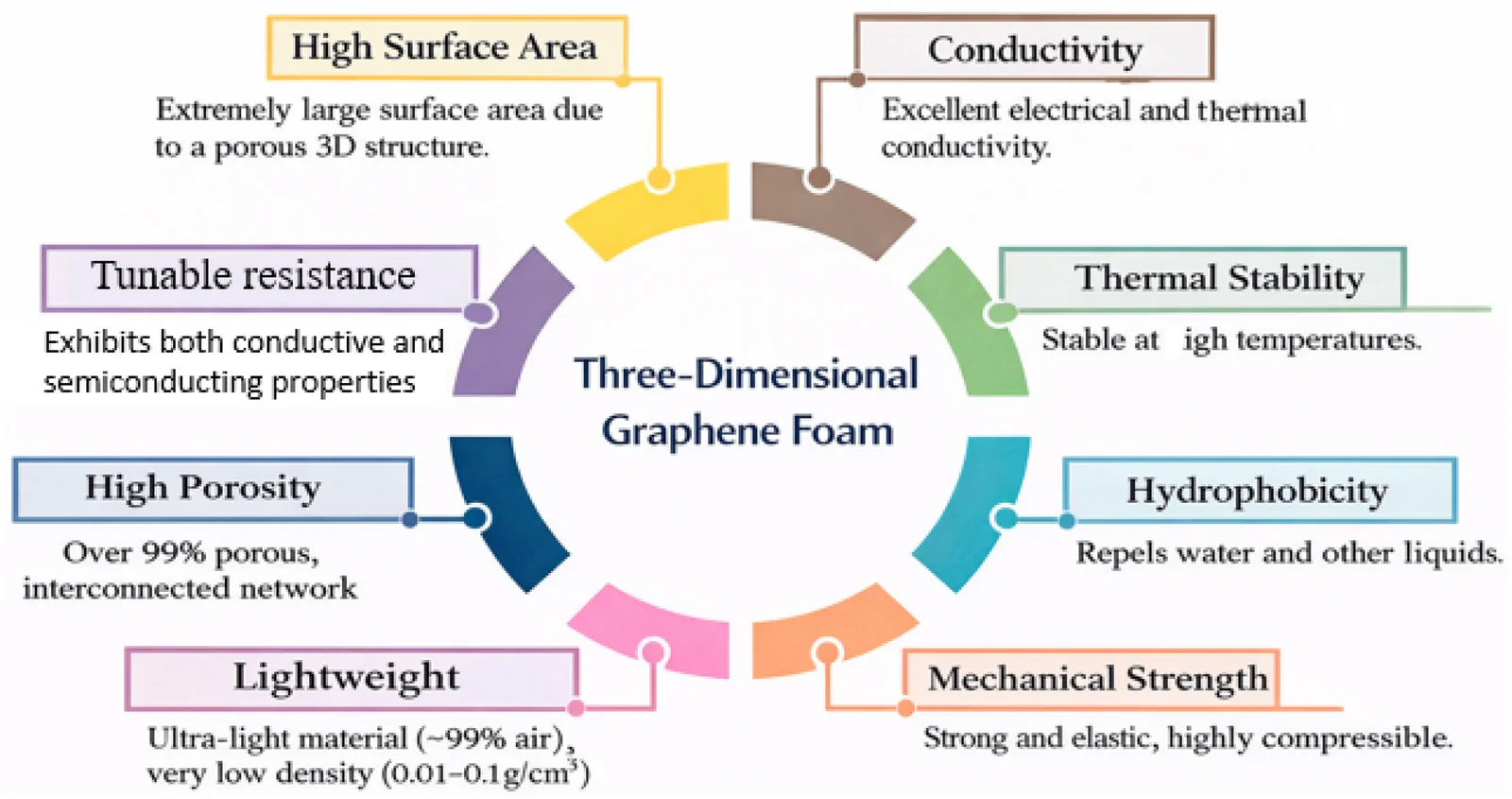
The properties of three-dimensional graphene foam.

Owing to this combination, 3D graphene foam has emerged as a promising platform for next-generation functional materials, finding utility in fields ranging from energy harvesting,^10^ electrochemical energy storage^11^ and flexible electronics to thermal management,^12^ environmental remediation, and bioengineering.^11,13^ The characterization of this material is therefore inherently multi-faceted, requiring detailed morphological, structural, chemical, thermal, and mechanical analyses to fully elucidate its performance metrics and application-specific behavior.^3,14,15^

Morphologically, techniques such as scanning electron microscopy (SEM) provide crucial insight into the architecture of the foam, which typically consists of an open-cell network with pore sizes spanning from a few micrometers to several hundred micrometers.^16^ This hierarchical porosity not only contributes to the material's ultralow density but also enhances electrolyte infiltration, mass transport, and catalytic accessibility. These features make 3D graphene foam highly suitable for electrochemical energy storage, catalyst support, and filtration membranes. Complementary characterization using Brunauer–Emmett–Teller (BET) surface area analysis quantifies the specific surface area and pore size distribution,^17^ parameters that directly influence diffusion kinetics, adsorption capacity, and electrochemical double-layer formation—critical factors in applications such as supercapacitors, batteries, and adsorbents, as shown in Fig. 2.^18^

**Fig. 2:**
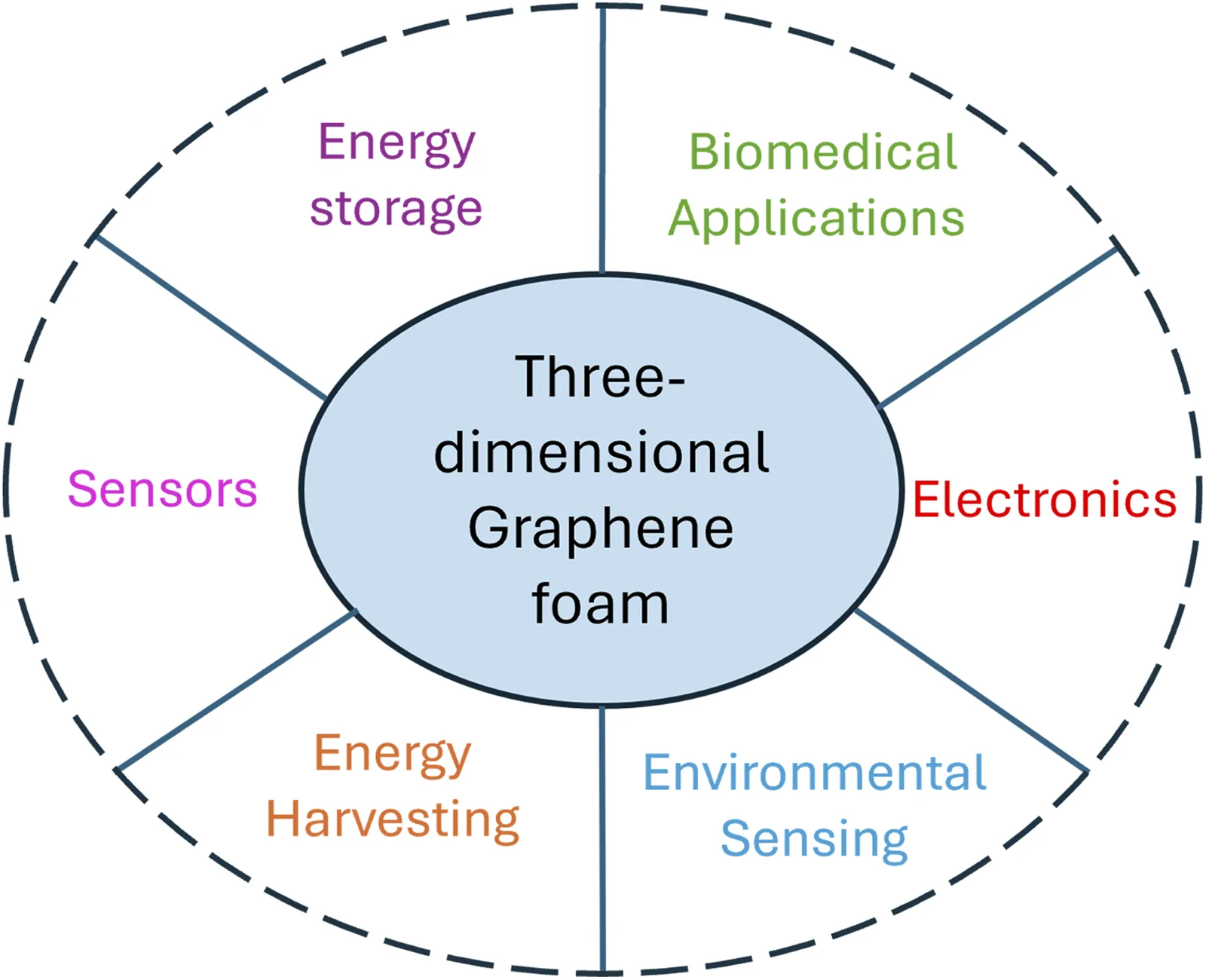
The primary applications of three-dimensional graphene foam.

Structurally, X-ray diffraction (XRD)^19^ and Raman spectroscopy^20^ serve as indispensable tools for probing the internal arrangement of graphene sheets within the foam. XRD provides information on crystallinity, interlayer spacing, and the degree of graphitization, all of which impact the foam's ability to conduct heat and electricity. Raman spectroscopy, with its sensitivity to defects and disorder, enables evaluation of the graphene framework through characteristic D, G, and 2D bands. The D/G intensity ratio is widely used to assess defect density, while the spectral shape of the 2D band offers insight into the number of graphene layers and stacking order.^21^ High-quality graphene foams typically exhibit a low D/G ratio and a well-defined 2D peak, reflecting minimal structural imperfections and strong interconnectivity—attributes that enhance both mechanical robustness and electrical transport.^3,18^

At the chemical level, X-ray photoelectron spectroscopy (XPS)^22^ and Fourier-transform infrared spectroscopy (FTIR)^23^ allow for the identification of surface functional groups, oxidation states, and heteroatom dopants. Chemical functionalities introduced during synthesis or post-processing can significantly modify the interfacial properties of graphene foam, altering its surface energy, wettability, chemical stability, and compatibility with electrolytes or polymeric matrices.^16^ Controlled functionalization may also enhance catalytic activity or facilitate anchoring of nanoparticles, further broadening the material's range of applications.

Mechanical and electrical characterization plays an equally critical role in evaluating the suitability of 3D graphene foam for structural and electronic devices. Compression testing reveals the foam's ability to withstand and recover from large strains, a behavior stemming from the intrinsic strength of the graphene framework and its cellular geometry.^24^ Many graphene foams demonstrate remarkable elasticity, fatigue resistance, and structural stability, making them promising candidates for flexible sensors, mechanical damping systems, and lightweight structural components.^25^ Electrical assessments using four-point probe measurements highlight the continuous conductive network inherent to the foam, resulting in high electrical conductivity and efficient charge transport.

Finally, the electrochemical performance of 3D graphene foam as an electrode material for supercapacitors has been extensively examined.^26^ There are a number of studies available in the literature displaying similar energy storage abilities, such as the study by Sun. Xiaoming, *et al.*, which described reduced graphene and carbon nanofiber hybrid electrodes. The electrodes were prepared by liquid-phase impregnation of cellulose paper in an RGO suspension, followed by freeze drying and activation with a reported specific capacitance of 30 µF cm^−2^.^27^ Similarly, a study by Ma. Xinlon and co-workers reported a normalized areal capacitance of 38 µF cm^−2^ where they used MgSO_4_-containing porous whiskers as templates and a S source. The S-doped carbon electrode produced at 600 °C displayed the highest areal capacitance values.^28^ These studies have used extremely complicated and energy intensive processes to produce active materials. The iGii's process is a low-energy electrically powered process, foregoing the use of mined metals and toxic chemicals. It utilizes 0.026 kWh per cm^2^ of the 3DG film, which can be as low as 9.3 kWh kg^−1^ compared to CVD, which can be 80.3 kWh kg^−1^, coupled with high level of scalability. This highly porous material has immense potential for hybridization with high energy materials such as metal oxides to improve performance even further.^29^ Therefore, overall, this method of producing active materials is both sustainable, cost-effective and scalable, with huge potential of enhancement in performance, further making it more desirable than currently employed active materials where conventional synthesis strategies are used. When employed as a freestanding electrode, graphene foam typically exhibits ideal electrical double-layer capacitor (EDLC) behavior,^30^ with cyclic voltammetry and galvanostatic charge–discharge analyses showing rectangular voltammograms and triangular profiles without significant pseudocapacitive contributions. Electrochemical impedance spectroscopy (EIS)^31^ further confirms low equivalent series resistance and rapid ion diffusion within the porous network, underscoring the material's excellent conductivity. Due to its EDLC-driven charge storage mechanism and minimal resistive losses, graphene-foam-based electrodes demonstrate outstanding rate capability, retaining a substantial proportion of their capacitance even as current density increases by an order of magnitude.^32^

This paper provides a description and analysis of these complementary characterization techniques, providing a holistic understanding of three-dimensional graphene foam and highlighting how its hierarchical architecture, defect landscape, chemical surface functionalities, and mechanical–electrical properties converge to dictate its multifunctional performance. Such insights are essential for optimizing synthesis parameters, tailoring properties for specific applications, and advancing the integration of 3D graphene foam into next-generation technological systems.

## 2. Results and discussion

### 2.1. SEM

Fig. 3 shows SEM images of the surface and cross-section of high defect (HD) and low defect (LD) graphene foam samples. To allow a direct comparison, images were acquired at the same magnifications for both samples. Additionally, multiple magnifications were used to better visualize the morphology of the graphene foam samples at the nanoscale. In both cases, they appear to be in the form of “crumpled” nanosheets, two-dimensional nanomaterials that have been intentionally folded or deformed into three-dimensional, nonplanar structures. This “crumpled” morphology offers advantages over flat nanosheets, including increased surface area, better dispersion in solvents, and improved functional properties. The increased surface area is particularly beneficial in applications such as catalysis, sensing, and energy storage, where it can lead to increased capacitance, conductivity, and catalytic activity.^3,14,33^ Furthermore, the unique structure of crumpled graphene oxide nanosheets enables the fabrication of highly sensitive and responsive sensors, making them attractive for a wide range of advanced technologies.^16^ The high defect GF sample exhibits a more inhomogeneous structure, characterized by loosely packed nanosheets and larger voids in both surface and cross-section images. The nanosheets appear to be more fragmented and irregular, suggesting higher resistance due to weaker electrical contact and discontinuity in the conduction paths. In contrast, the low defect GF exhibits more compact and uniform morphology. The surface appears denser with more consistent and interconnected structures, while the cross-section images show a more uniform granular texture, suggesting enhanced conductivity between nanosheets. This morphological improvement is consistent with a better electrical performance observed in the *I*–*V* measurements, as the more uniform conduction paths lead to more efficient charge transport, as shown in Fig. 4.

**Fig. 3:**
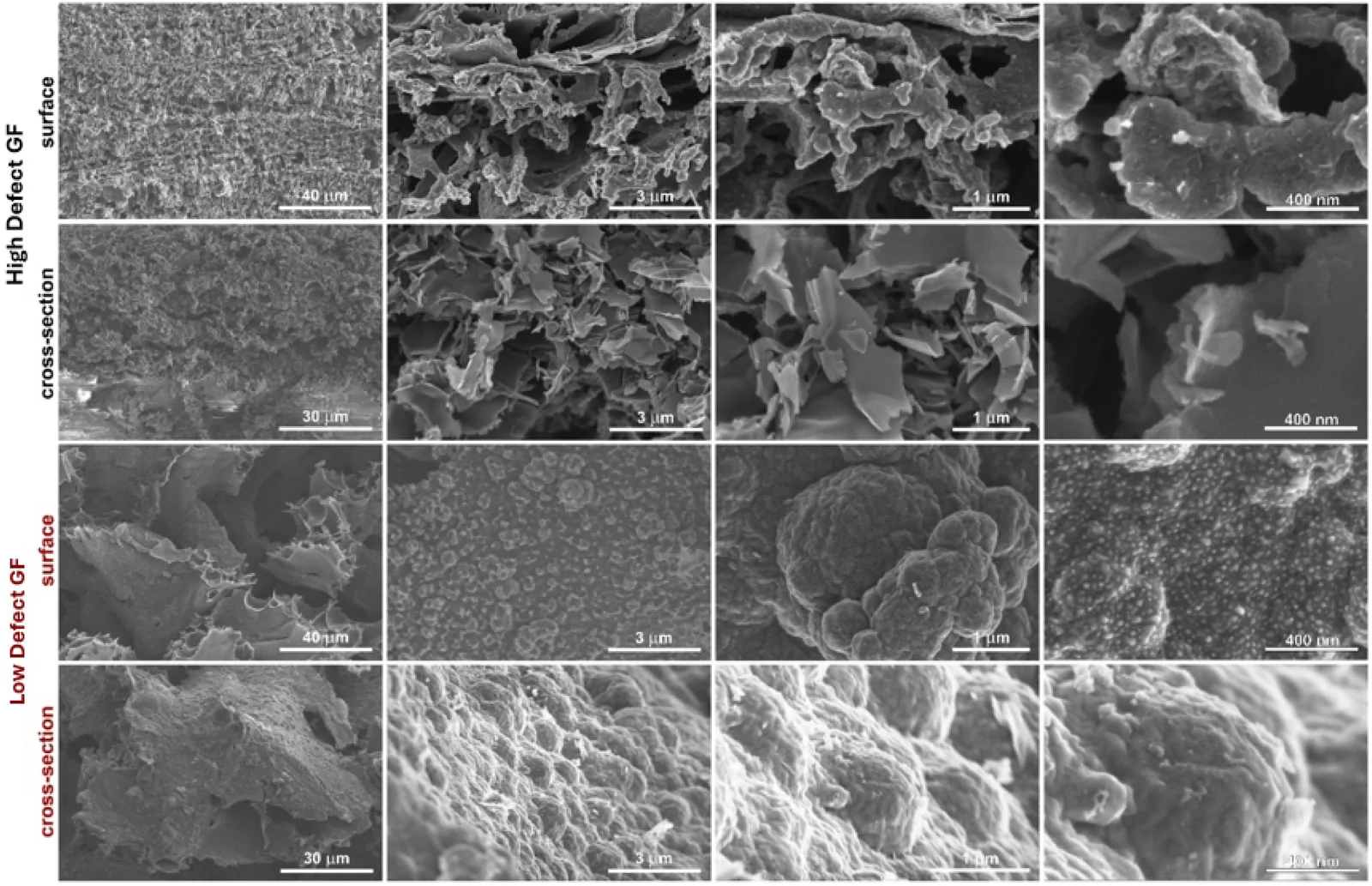
SEM images at various magnifications of high and low defect graphene foam.

**Fig. 4:**
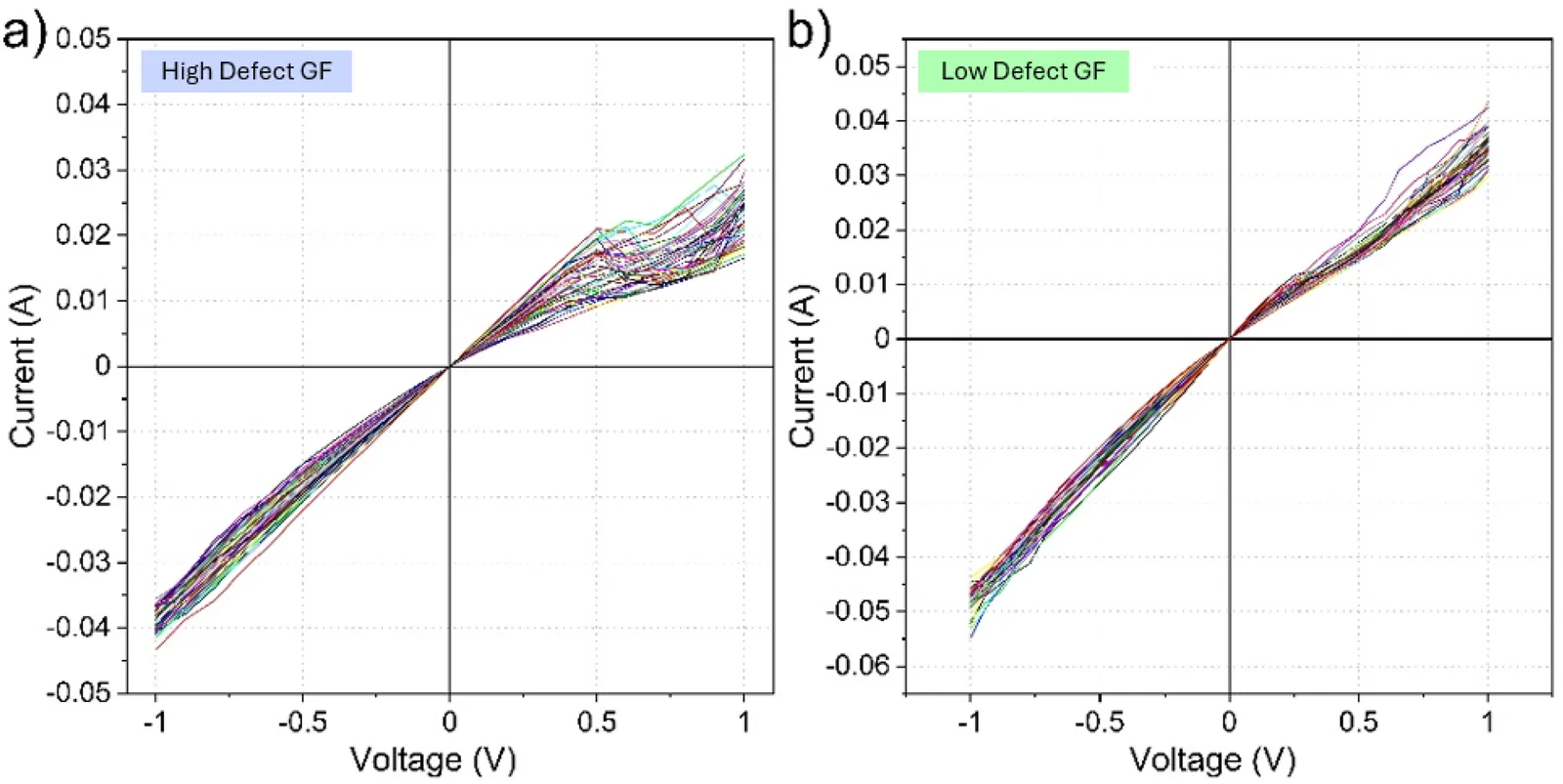
Current–voltage characteristics of: (a) high and (b) low defect graphene foam.

### 2.2. Current–voltage characteristics and optical microscope imaging

The current–voltage characteristics for high and low defect GF are shown in Fig. 4. In both cases, the voltage sweep mode was adopted in the range of −1 to 1 V. The characteristics showed linear behavior, indicating ohmic conductivity. The HDG foam exhibited higher resistance, as evidenced by the lower current response over the entire voltage range. In comparison, the LDG foam showed a steeper slope and higher current values, consistent with improved electrical conductivity. This is further proven when the sheet resistance for both materials was calculated using 50 *I*–*V* measurements, with HDGF measuring at 32.9 ± 3.7 Ω sq^−1^ and the LRGD measuring at 26.1 ± 1.5 Ω sq^−1^. The reduced scattering in the *I*–*V* curves of the low defect graphene foam may suggest better uniformity of the GF or quality of the electrodes deposited on top of the GF.

As demonstrated by the images captured using a digital camera, there is a clear visual distinction between the two samples (Fig. 5). The high defect GF (Fig. 5(a)) exhibits a more uniform and deep black appearance, indicative of higher light absorption and possibly a more compact or homogeneous surface. On the other hand, the low defect GF (Fig. 5(b)) shows a lighter, more metallic-grey tone with visible texture lines, suggesting a rougher surface and greater light scattering – this is in line with optical reflectance measurements displayed in Table 1, where the high defect GF has a lower average reflectance (0.028%) over the visible region as compared with the low defect GF (0.153%). These differences in visual appearance are likely due to differences in surface morphology, which are also visible in optical microscope images. The low defect GF appears to be more textured at the macroscale, which may contribute to its lower electrical resistance or changed optical properties, such as light absorption or reflection.

**Fig. 5:**
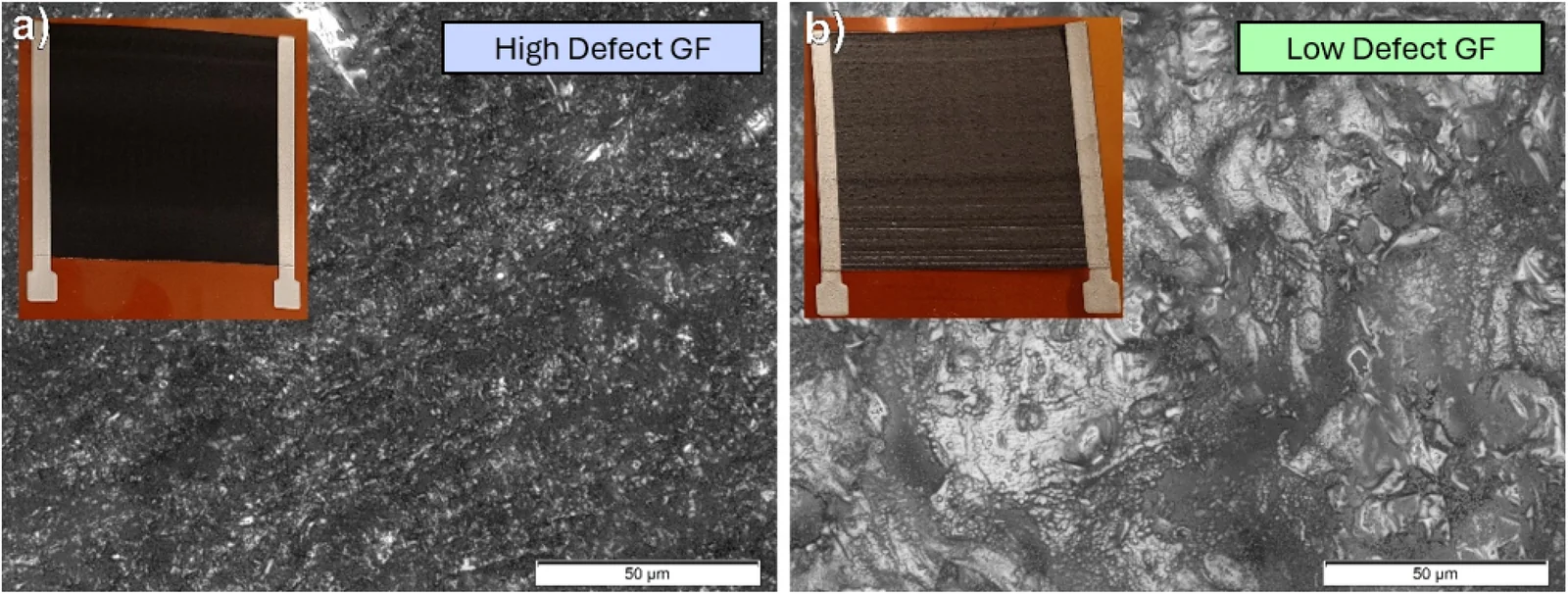
Optical microscope images and corresponding photographs (insets) of: (a) high and (b) low-resistivity graphene foam.

**Table 1:** Average values for optical transmittance and reflectance (350–1700 nm) and optical transmittance and reflectance (2.5–25 µm) for sample-on-Kapton

Sample (on Kapton)	Visible (350–1100 nm)		Mid-wave and long-wave infrared (2.5–25 µm)	
	Short-wave infrared (1100–1700 nm)			
	*T*_av_ (%)	*R*_av_ (%)	*T*_av_ (%)	*R*_av_ (%)
High defect GF	−0.0034 ± 0.01	0.0280 ± 0.01	−0.0042 ± 0.01	0.1032 ± 0.01
Low defect GF	0.0145 ± 0.01	0.1533 ± 0.01	0.0037 ± 0.01	0.3207 ± 0.01

### 2.3. AFM

Fig. 6 combines AFM and SEM measurement results to compare the surface topography of high and low defect graphene foam samples. The AFM images revealed significant differences in nanoscale roughness between the two samples. The high defect GF shows a pronounced surface roughness, with a maximum surface height of over 18 nm. The surface appears irregular, with larger grain features, which correlates well with the SEM image. On the other hand, the low defect GF exhibits a much smoother and more uniform surface in the AFM image, with a maximum surface height of *ca.* 4.5 nm. The nanoscale texture is finer and more compact, showing small grains at the surface, which also align well with the SEM observations. This smoother morphology is consistent with the SEM image, where the foam surface shows densely packed, granular structures, suggesting improved contact between the nanosheets. Surface topography investigations seem to show that the low defect GF has significantly lower nanoscale roughness. These morphological improvements likely contribute to its enhanced electrical properties, as shown by the lower resistance values.

**Fig. 6:**
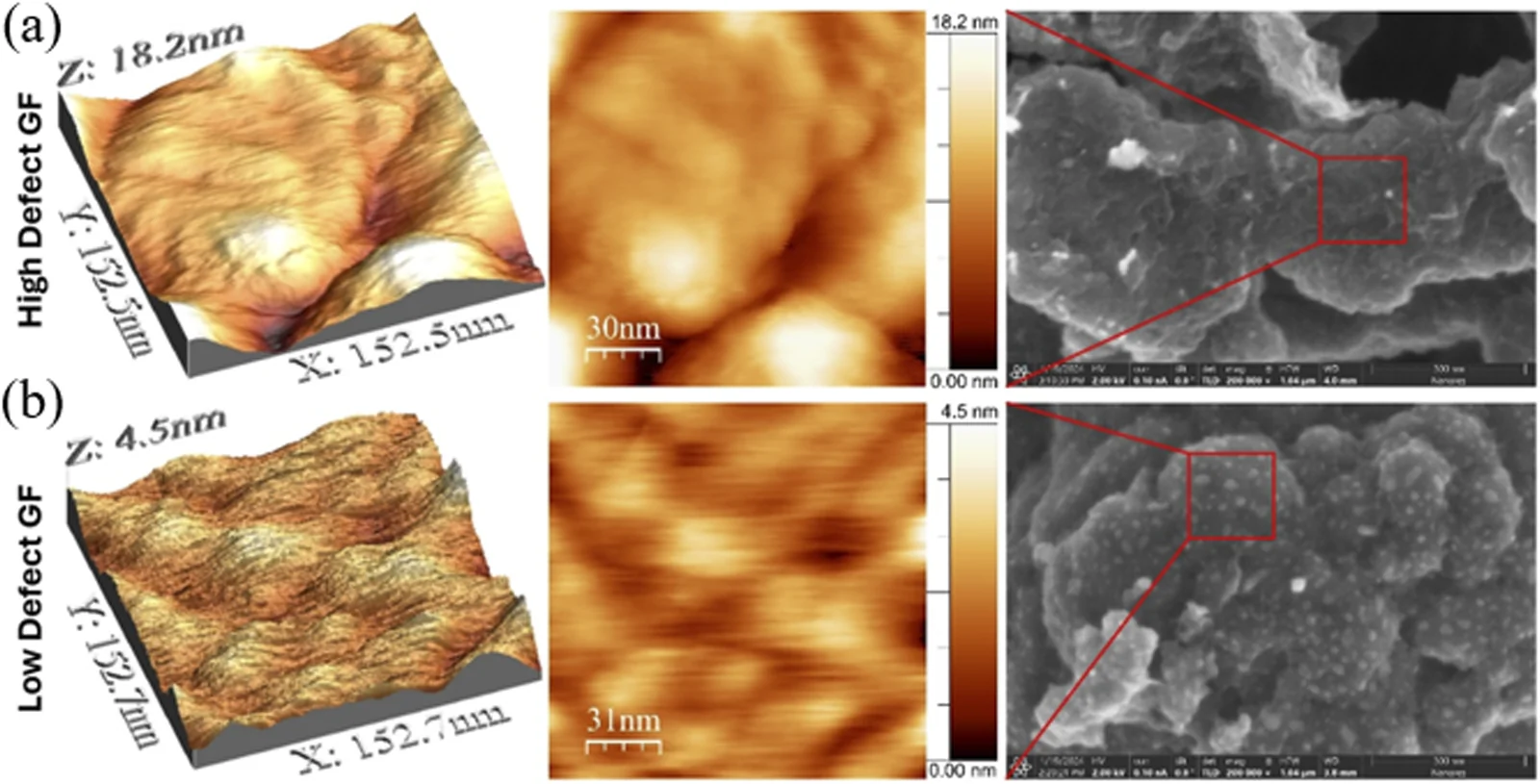
AFM images of the surface of: (a) high and (b) low defect graphene foam.

### 2.4. EDS and XPS measurements

Material composition determined by energy-dispersive X-ray spectroscopy (EDS) and surface chemical state analysis performed using X-ray photoelectron spectroscopy (XPS) confirmed that both graphene foam samples are primarily composed of carbon (Fig. 7). The EDS spectra (Fig. 7(a) and (b)) revealed dominant peaks corresponding to carbon and oxygen, along with minor peaks attributed to nitrogen (N), calcium (Ca), and phosphorus (P). These very small peaks may indicate either specific synthesis methods or trace impurities present in both samples. The XPS survey scans (Fig. 7(c)) further confirmed the presence of carbon (C 1s and C KLL) and oxygen (O 1s, O 2s, and O KLL), with small peaks corresponding to N, Ca, and P, in agreement with the EDS results. Notably, the intensity of the O 1s peak is lower in the low defect GF compared to the high defect GF, suggesting a reduced oxygen content or a difference in surface functional groups.

**Fig. 7:**
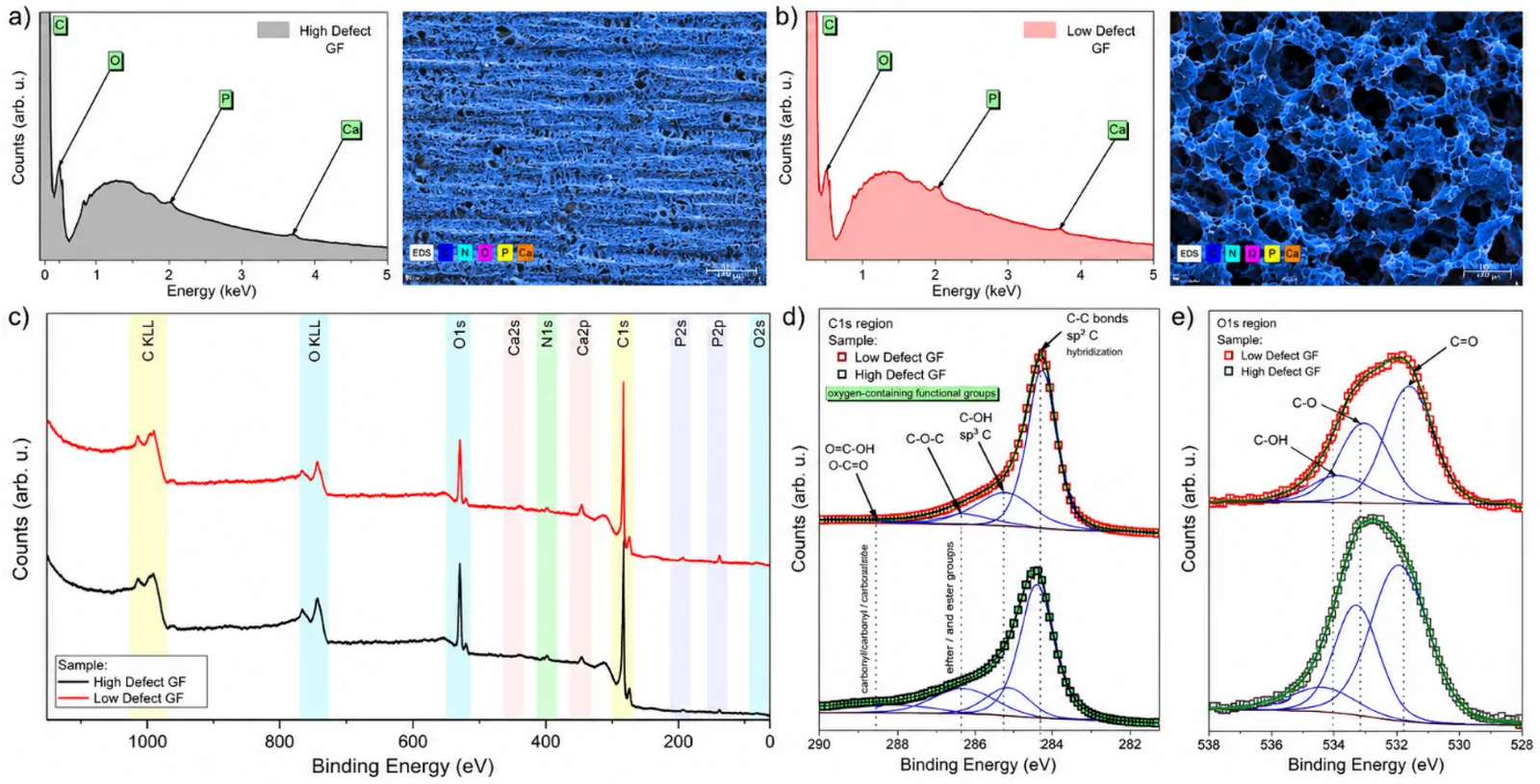
Chemical state of investigated samples: EDS spectrum and map of distribution of elements in (a) high and (b) low defect graphene foam, (c) XPS survey scan and high-resolution scans of (d) C 1s region and (e) O 1s region.

High resolution C 1s core-level spectra, resembling reduced graphene oxide, were deconvoluted into four peaks for both samples (Fig. 7(d)). The peak at approximately 284.4 eV was assigned to carbon atoms with sp^2^ hybridization, while the one at *ca.* 285.3 eV to sp^3^ hybridization. The relative area of the sp^2^ peak was 53.9% for high defect GF and 62.6% for low defect GF. According to Wei *et al.*^18^ an increase of sp^2^-hybridized carbon enhances electrical conductivity. Consistent with this, the low-defect GF, which exhibited higher sp^2^ hybridization content, also showed lower electrical resistance compared to high defect GF. Furthermore, additional peaks at *ca.* 286.3 eV and 288.5 eV were assigned to hydroxyl/epoxy groups and carbonyl/carboxyl functionalities, respectively. These results are in good agreement with previous literature reports.^11,24^ The O 1s spectra for both samples were deconvoluted into three peaks (Fig. 7(e)) located at approximately 531.8 eV (C=O bonds), 533.2 eV (C–O bonds), and 534.1 eV (C–OH bonds), further confirming the presence of oxygen-containing functional groups on the graphene foam surfaces.

### 2.5. Wettability

Wettability measurements showed that the high defect GF was super hydrophilic, with a contact angle (CA) of approximately 14°, while the low defect GF was hydrophilic, exhibiting a CA of *ca.* 72°, as shown in Fig. 8. Moreover, water droplets on the high defect GF surface tended to spread very fast, decreasing the CA to nearly zero degrees within a few seconds. It is assumed that these differences in wettability of both tested samples are likely related to changes in surface morphology and active surface area. In particular, the larger voids between nanosheets in the low defect GF may cause the rapid spreading of water droplets.

**Fig. 8:**
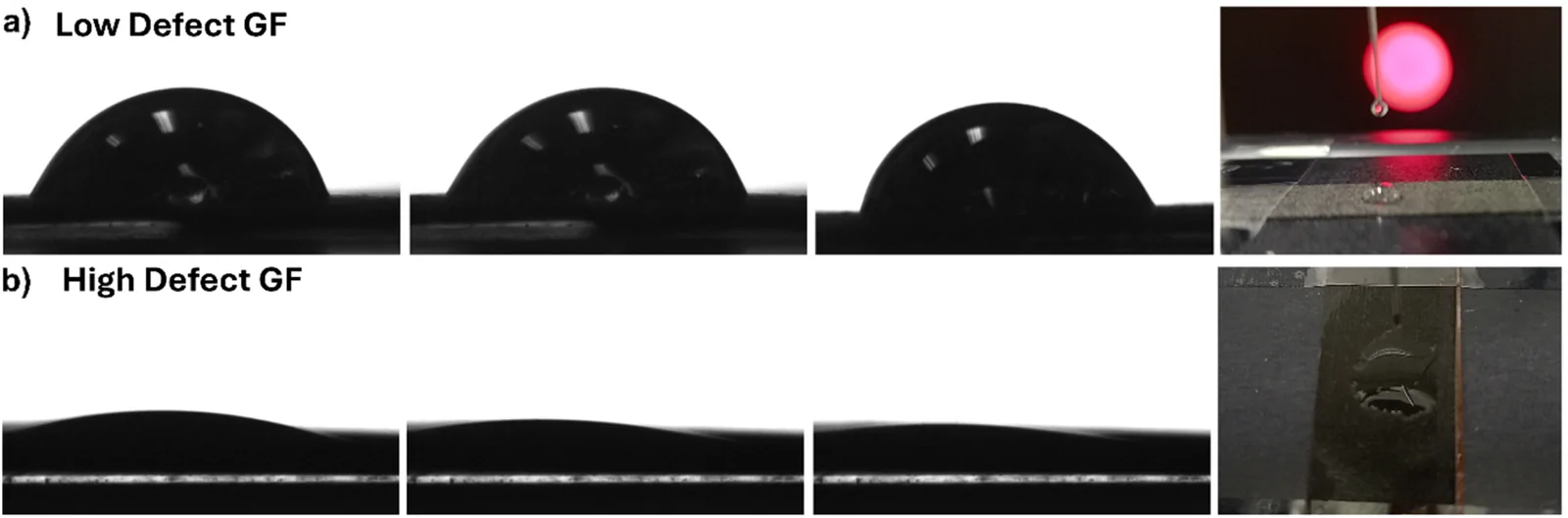
Wettability measurements results showing 3 water droplets on the surface of: (a) low and (b) high defect graphene foam.

### 2.6. Raman spectroscopy

Fig. 9(a) shows the Raman spectrum of high defect graphene plotting Raman shift *versus* intensity with 3 peaks represented as D, G and 2D peaks. This measurement is conducted using a 532 nm laser due to strong resonance with graphene's electronic structure, leading to a high Raman signal and good resolution of features. The D peak at 1349 cm^−1^ and the G peak at 1586 cm^−1^ are in the first order Raman spectrum, representing disorder and the graphite signature of carbon, respectively. The 2D peak at 2667 cm^−1^ is part of the second order Raman spectrum, this peak is also sometimes known as G′, as it is the second harmonic of the phonon density.^20^ In Fig. 9(a), the G peak is at a higher intensity than that of the D peak meaning the high defect GF is more carbon based with fewer defects present in the material.

**Fig. 9:**
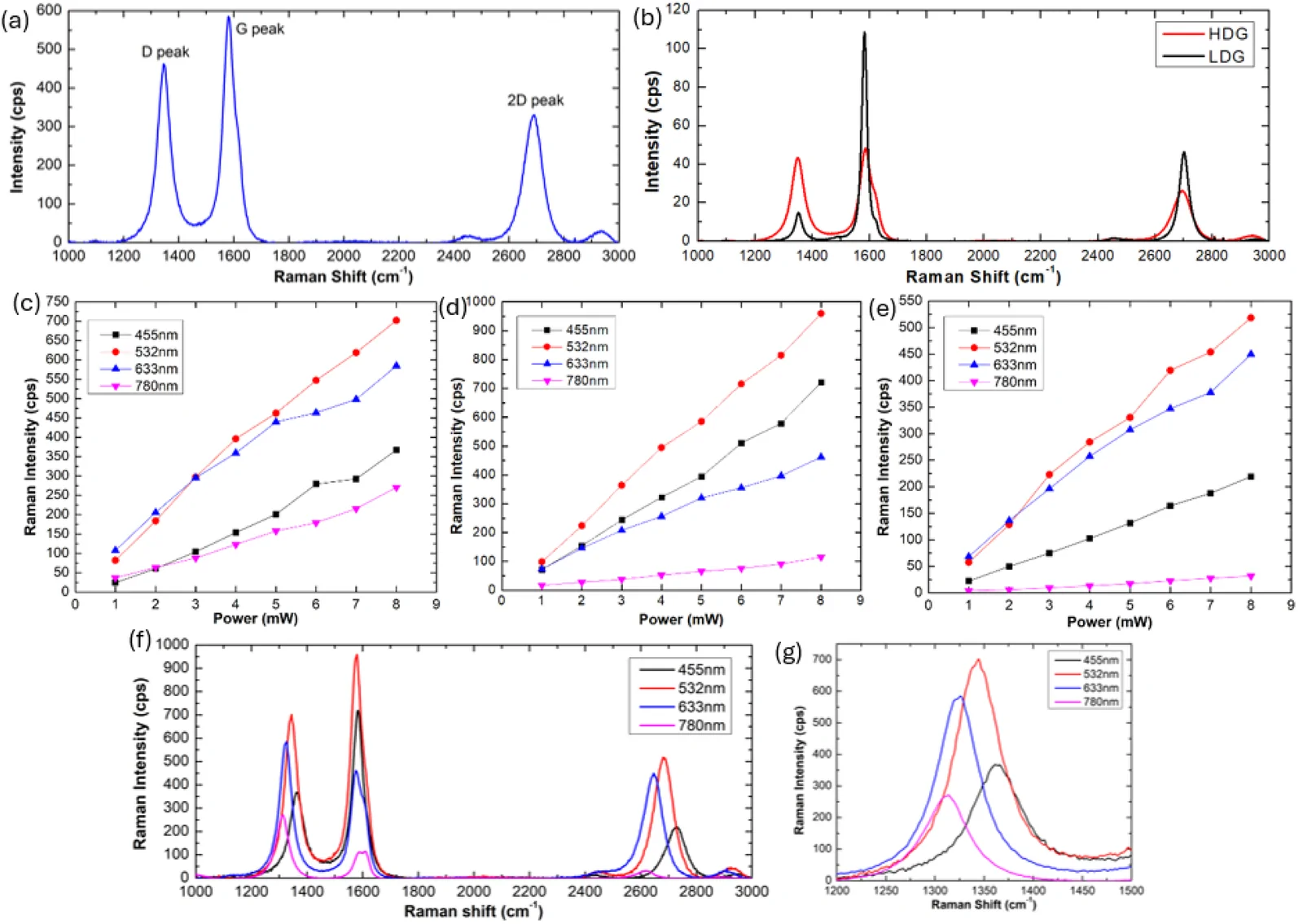
(a) Different peaks in a Raman spectrum for HGGF at 532 nm and 0.5 mW, (b) comparison of peaks for LDGF and HDGF foam at 532 nm and 0.5 mW. Comparison of intensity *vs.* power for (c) D, (d) G and (e) 2D peak positions; (f) comparison of 4 wavelengths and (g) comparison of the D peak only.

Fig. 9(b) shows a comparison of the high and low defect graphene foam samples using an excitation wavelength of 532 nm at 0.5 mW. The high defect GF presents a 0.9 *I*_D_/*I*_G_ intensity ratio, which is 0.8 higher than that observed in low defect GF with an *I*_D_/*I*_G_ ratio of 0.1, exhibiting a more drastic change in the electronic properties of both materials obtained during the synthesis. One of the main differences between the materials is their structure; the high defect graphene material has a more porous structure whereas the low defect graphene is more compact, meaning that there is less space for particles to move, as shown in Fig. 3.

Power measurements were taken in the mW range since high-powered laser irradiation onto a carbon-based sample can change and/or damage the surface of the material. Fig. 9(c)–(e) present each specific peak in the high defect graphene spectrum over a range of wavelengths from 455 nm to 780 nm. Across all three figures, the optimal wavelength for the high defect material is 532 nm; this is due to the low fluorescence background for carbon-based materials at approximately 500 nm.^35^ From Fig. 9(c) and (e), the D peaks are related showing the same intensity peaks to be higher at 633 nm than at 455 nm; this means there is a higher number of defects present in the material at 633 nm compared to that at 455 nm, which shows a higher value of graphite in the G peak position (Fig. 9(d)).

Fig. 9(f) shows the comparison of all four excitation wavelengths measured at 0.5 mW for the high graphene foam. Fig. 9(g) shows specifically the D peak in the high defect GF as a function of the excitation wavelengths, showing a clear ‘optical switch’ behavior happening when the material is irradiated with light below and above 532 nm, respectively. In the first investigation of the Raman spectrum of graphite, Tuinstra and Koenig^36^ related the D peak to the zone-centre phonon mode which is seen in single crystalline graphite but isn't Raman active.^37^ However polycrystalline materials like high defect graphene foam have disorder in their materials that allows them to be Raman active. It is known that the D peak is in the phonon density of states distribution, meaning that the photon energy varies with the excitation wavelength used.

### 2.7. Optical properties

In Fig. 10 the optical transmittance and reflectance of the high and low defect GF were found to be close to zero throughout the 350 nm to 25 µm wavelength range of the electromagnetic spectrum.

**Fig. 10:**
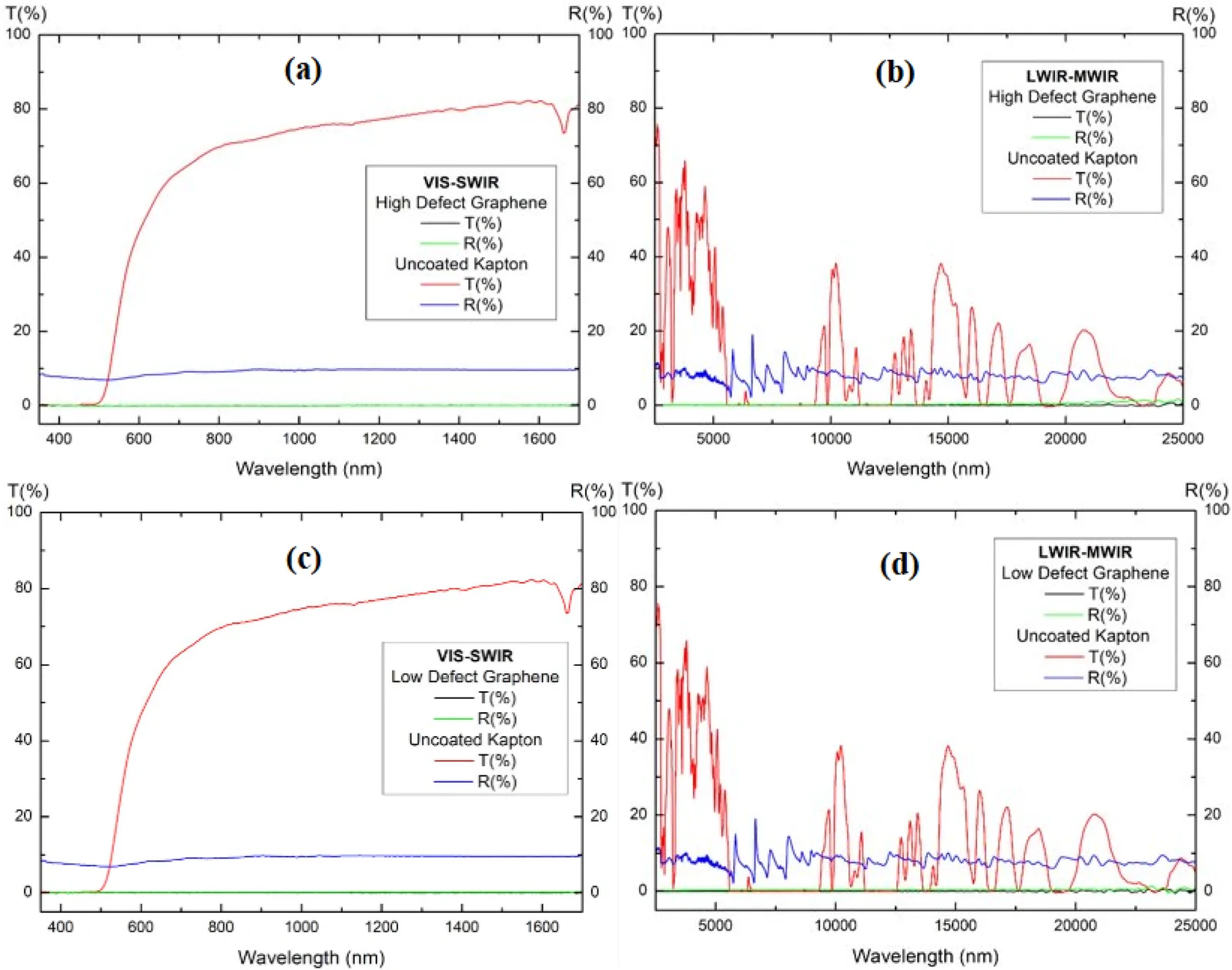
Transmittance and reflectance plots for HDG on Kapton; (a) visible/SWIR and (b) LWIR/MWIR, and LDG on Kapton; (c) visible/SWIR and (d) LWIR/MWIR.

The uncoated Kapton transmittance and reflectance curves are shown by the red and blue curves respectively, and the coated GF on Kapton is shown by the black and green curves, which closely overlay around 0%. The curves are specified in the inset plots within Fig. 10.

This suggests that the coating material has a high optical density due to optical absorption and light trapping. GF may find applications as a broadband optical detector material or as an absorber coating for stray light in optical systems.

Stray light generally refers to unwanted light that was not intended in the design of an optical system for both imaging and non-imaging based optical systems. Such unwanted light can arise due to optical scattering, diffraction and thermal emission and gives rise to optical imaging artefacts such as lens flare, light leakage, scattering, ghosting and veiling glare^38^ which can have important implications for optical systems in automotive safety, aerospace perception, medical accuracy and augmented/virtual reality experiences.

Methods of reducing the effects of stray light in an optical system include the use of an optical baffle^39^ which is an opto-mechanical component used to block light. It is important that optical baffles block the full wavelength range of interest and do not reflect a large percentage of the light.

The optical transmittance and reflectance of high and low defect GF on Kapton were measured in the visible (VIS) and short-wave infrared (SWIR) regions using an Aquila NKD-8000 spectrophotometer (350–1650 nm)^40^ and in the mid-wave infrared (MWIR) and long-wave infrared (LWIR) regions using a Nicolet iS50 Fourier Transform Infrared Spectrometer (2500–25 000 nm)^41^. Fig. 10(b) show the VIS and SWIR optical transmittance and reflectance spectra for high and low defect GF. The optical transmittance of an uncoated sheet of Kapton was also measured and is overlaid in the plot for comparison. Both the transmittance and reflectance are reduced to almost zero by application of the GF coating. The case is true for both the high and low defect GF from the *vis* through to the LWIR region.

Table 1 shows the average transmittance (*T*_av_) and average reflectance (*R*_av_) over this range.

The values presented in Table 1 have been extracted from the measured data presented in Fig. 10.

For both the high and low defect GF, the *T*_av_ measurement value is close to zero, suggesting that almost no VIS and SWIR light passes through the sample. The case is similar for *R*_av_ for high defect GF. The *R*_av_ for the low defect GF is slightly higher, in the 0.14% to 0.16% range which could be due to the difference in the microstructure between the two coating materials, as the low defect GF material appears less porous which would lead to a higher specular reflection as compared with the high defect GF.

In the MWIR/LWIR regions (see Table 1), *T*_av_ for the high and low defect GF is similar to the *T*_av_ for the VIS/SWIR regions and is close to zero. *R*_av_ for both materials is slightly higher in the MWIR/LWIR region as compared to the VIS/SWIR region. The gap in the optical measurements is due to the limited spectral ranges of the spectrometer equipment used.

In the visible and NIR regions, both the transmittance and reflectance were measured relative to an uncoated JGS3 fused SiO_2_ reference standard and corrected for using the known values of JGS3 glass transmittance and reflectance (*T*_av_ = 93.13% and *R*_av_ = 6.66% over the region).

In the SWIR and LWIR regions, the transmittance was measured relative to a no-sample atmosphere background reference (*T*_av_ = 100% over the region) and the reflectance was measured and corrected for relative to a gold coated glass disk reflector optic (*R*_av_ = 98% over the region). The measured values were close to zero; however, it is unknown how much of this is direct black body absorption and how much is due to scattering (due to the sample's porous structure).

These differences in optical properties are most likely due to differences in the microstructure of the materials, particularly the density and size of the pores. The observed low optical transmittance and reflectance suggest that GF is an excellent material for absorbing and trapping light over a broad spectral range and may have useful applications in reducing stray light in optical systems and other applications where broadband optical absorption is important.

### 2.8. Electrochemical characterization

The electrochemical properties of prepared samples were examined using a standard three electrode test cell setup. Both CV and GCD measurements were carried out in the potential window of 0–0.8 V for the electrode comprising low defect (LR) and high defect (HR) samples. All the measurements were carried out under ambient conditions (at room temperature and pressure) using 2 M Na_2_SO_4_ (142.04 g mol^−1^ molarity) aqueous electrolyte. Both samples based on three-dimensional graphene foam displayed electric double layer capacitive behavior as shown by CV and GCD profiles in Fig. 11(a)–(d).

**Fig. 11:**
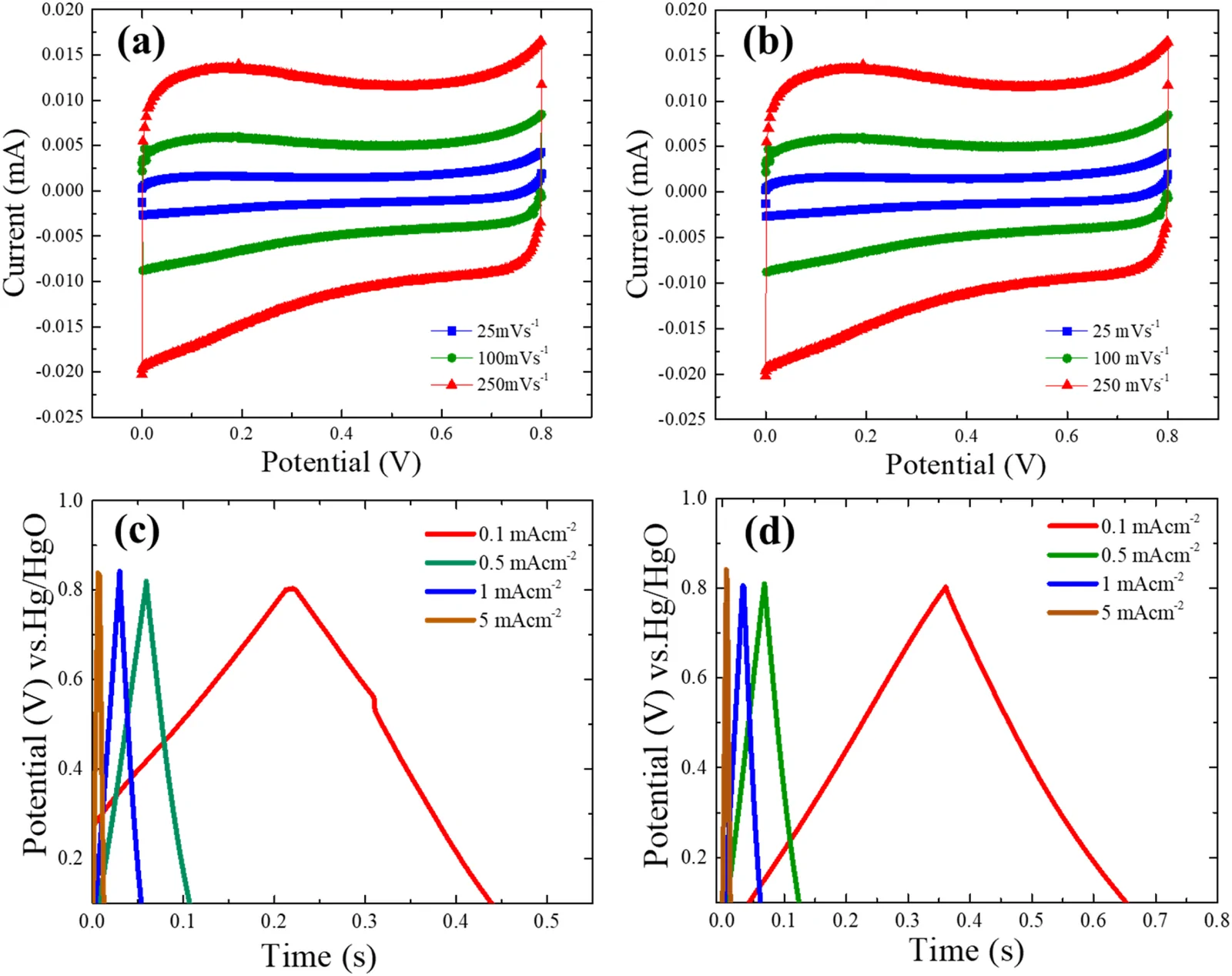
(a) Cyclic voltammograms of the LDGF sample, (b) cyclic voltammograms of the HDGF sample, (c) GCD profile of the LDGF sample and (d) GCD profile of the HDGF sample.

The CV curves of LDGF and HDGF samples are displayed in Fig. 11(a) and (b), respectively, at scan rates of 25, 100 and 250 mV s^−1^. The results exhibit an ideal electric double layer behavior with cyclic voltammograms being symmetrical, without any redox peaks and nearly rectangular in shape even at much higher scan rates. Retention of rectangular shapes at higher scan rates demonstrates the sample's excellent rate capability due to the highly conductive active material and physical charge storage mechanism.^26^ This can also be confirmed from Table 2 where the drop in capacitance is particularly small when compared with similar materials considering the increase in current density and drop in capacity retention^34,42^ GCD measurements were also carried out at current densities of 0.1, 0.5, 1 and 5 mA cm^−2^ to analyze the capacitive behavior and to measure areal capacitance of the samples. The GCD profiles of both samples are shown in Fig. 11(c) and (d). Typical triangular shaped GCD profiles at all current densities signify low resistance electric double layer behavior which is also in good agreement with CV analysis.^43^

**Table 2:** Measured areal capacitances of LDGF and HDGF samples at different current densities

Sample	Capacitance (µF cm^−2^)			
	Current density (mA cm^−2^)			
	0.1	0.5	1	5
LDGF	32	31	30	30
HDGF	38	36	36	34

Table 2 shows that the HDGF sample has superior capacitive performance with the highest areal capacitance of 38 µF cm^−2^ at a current density of 0.1 mA cm^−2^ which is around 16% higher than that of the LDGF sample. As discussed earlier, the porous structure is considered to be the key contributor towards electric double layer charge storage. SEM micrographs in Fig. 3 show that HDGF has much higher level of porosity compared with LDGF; therefore, it can be presumed that the higher capacitance of the HDGF sample can be due to its well-developed porous structure with a high level of porosity. The detailed study of Table 2 shows that there was around 6% drop in storage capacity for the LDGF sample. However the drop in capacity retention was higher around 11% for HDGF when the current density was increased by 50 times. The higher drop in capacity for the HDGF sample can be credited to more pronounced resistive behavior which can also be confirmed by comparative Nyquist plots from electrochemical impedance spectroscopy (EIS) analysis. The calculated specific capacitance as a function of the discharge current density has been plotted in Fig. 12(a). It is apparent that even at a much higher current density of 5 mA cm^−2^, HDGF and LDGF samples retained nearly 89% and 94% of the initial capacitance, respectively, displaying outstanding rate capability making these materials highly suitable for high power applications.

**Fig. 12:**
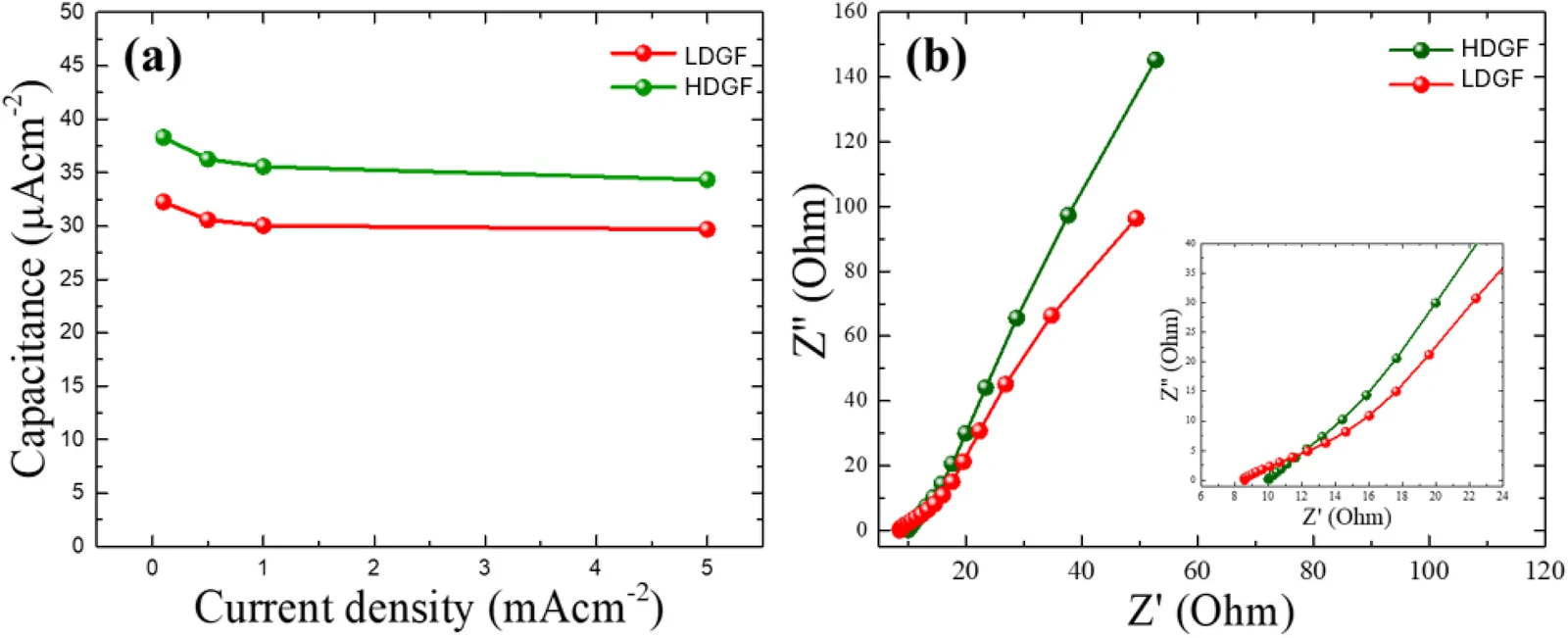
(a) Specific capacitance as a function of the discharge current density and (b) comparative EIS spectra of HDGF and LDGF samples.

Comparative Nyquist plots of LDGF and HDGF samples are shown in Fig. 12(b). The Nyquist plot has three main frequency regions *i.e.*, low, mid and high. The high frequency segment of EIS spectra signifies the combine series resistance (*R*_S_) of an electrolyte, current collector and separator, whereas the mid frequency region offers information about double layer capacitance. Finally, the low frequency part of the curve indicates the capacitive behavior attributed to the adsorption of electrolyte ions on the electrode surface.^44^ Graphene foam-based samples are highly conductive, and, as anticipated, the LDGF sample displayed even smaller *R*_S_ which can be estimated from the intercept along the *x*-axis (shown in the inset) in the high frequency region which is in a single digit for both samples (between 8 and 10 Ω).^45^ LDGF has an estimated resistance value of around 8 Ω, whereas HDGF has a slightly higher value of around 10 Ω. This is in good agreement with interfacial wettability analysis where both samples are hydrophobic with LDGF foam being superhydrophobic, therefore offering good wettability to aqueous solutions. This is followed by excellent capacitive behavior demonstrated by the linear profile and an ideal slope angle of around 45° to the real axis in the low frequency region. EIS analysis confirms that the graphene foam sample is highly conductive with low charge resistance and efficient reaction kinetics.

### 2.9. Summary of the results in table format

Table 3 shows a summary of all measured parameters from the results above with a side by side comparison of High Defect Graphene Foam (HDGF) and Low Defect Graphene Foam (LDGF).

**Table 3:** Property table comparing HDGF and LDGF

Material properties	HDGF	LDFG
Sheet resistance (Ω sq^−1^)	32.9 ± 3.7	26.1 ± 1.5
Surface roughness (nm)	18	4.5
Contact angle (°)	14	72
Defect ratio, *I*_D_/*I*_G_	0.9	0.1
Visible (350–1100 nm)	−0.0034 ± 0.01	0.0145 ± 0.01
Short-wave infrared (1100–1700 nm), *T*_av_ (%)		
Visible (350–1100 nm)	0.0280 ± 0.01	0.1533 ± 0.01
Short-wave infrared (1100–1700 nm), *R*_av_ (%)		
Mid-wave and long-wave infrared (2.5–25 µm), *T*_av_ (%)	−0.0042 ± 0.01	0.0037 ± 0.01
Mid-wave and long-wave infrared (2.5–25 µm), *R*_av_ (%)	0.1032 ± 0.01	0.3207 ± 0.01
Current density (0.1 mA cm^−2^)	38	32
Capacitance (µF cm^−2^)		
Current density (0.5 mA cm^−2^)	36	31
Capacitance (µF cm^−2^)		
Current density (1 mA cm^−2^)	36	30
Capacitance (µF cm^−2^)		
Current density (5 mA cm^−2^)	34	30
Capacitance (µF cm^−2^)		

## 3. Experimental section

### 3.1. Methodology

Current–voltage (*I*–*V*) measurements were conducted using a Keithley 4200-SCS semiconductor characterization system equipped with a Cascade MicroTech probe station featuring a thermal chuck heater.

Optical microscope images were acquired using an Olympus BX51 with a magnification of 500×. Elemental analyses and high-resolution images at the nanoscale were obtained with the use of a FEI Helios Xe-PFIB field-emission scanning electron microscope (FE-SEM) equipped with an energy dispersive X-ray spectroscopy (EDS) detector (Bruker). An UHV VT AFM/STM Omicron atomic force microscope working under ultra-high vacuum conditions in the contact mode was used to measure surface topography and to analyze the roughness of the graphene foam samples. The chemical state of the surface was verified using X-ray photoelectron spectroscopy (XPS) measurements employing a Specs Phoibos 100 MCD-5 hemispherical analyzer with a Specs XR-50 X-ray source. Wettability was assessed by water contact angle (CA) measurements performed with a Attention Theta Lite optical tensiometer.

Raman spectroscopy was performed using a confocal Thermo Scientific Raman microscope DXR equipped with four excitation wavelengths: 455 nm, 532 nm, 633 nm, and 780 nm. All measurements were conducted in backscattering geometry at room temperature. Samples were prepared by placing small sections of the graphene foam onto clean substrates to provide a low-background reference and ensure mechanical stability during analysis. To minimize laser-induced heating or structural modification, the incident power at the sample surface was carefully controlled for each excitation wavelength. The applied power did not exceed 8 mW for any of the lasers. Spectra were acquired using several short accumulations (5–10 scans, 5–30 s each) rather than a single long exposure to avoid thermal drift and facilitate cosmic-ray removal.

For the optical transmittance and reflectance measurements in the infrared region, a Thermo Fisher Nicolet iS50 Fourier Transform Infrared spectrometer was used. The system configuration employed a deuterated triglycine sulfate potassium bromide (DTGS KBr) detector and KBr beamsplitter. Scans were recorded at 8 cm^−1^ resolution, and data were averaged over 16 scans. Scans were recorded over a wavelength range of 2.5 µm to 25 µm. For optical measurements in the visible region, an Aquila NKD8000 spectrophotometer was used. Over the 350 nm to 1100 nm wavelength range, Si detectors were used and over the 1100 nm to 1700 nm wavelength range, InGaAs detectors were used. Scans were recorded at 2 nm resolution with no averaging.

The electrochemical performances were evaluated using a Biologic VSP-200 in a typical three-electrode setup. The reference electrode used was potassium chloride, the counter electrode was platinum foil, and the working electrodes were high and low defect graphene, with 0.8 M sodium sulfate as an electrolyte.

### 3.2. Fabrication of the material

The 3DG material used in this work is a 3DGii™ material grown by a patented process by Integrated Graphene Ltd.^5–8^ The 3DG growth process is based on a catalyst-free, direct-write graphene synthesis route that eliminates the need for metal catalysts and post-growth transfer steps, thereby preserving the structural integrity of both the graphene network and the underlying flexible substrate. Growth is carried out directly on the Kapton® HN polyimide substrate under controlled conditions that promote the formation of a three-dimensional, porous graphene architecture. Importantly, the fabrication route is compatible with large-format substrates, enabling scalable production of 3DG films up to A4 size using a low-cost and reproducible process. Direct growth on the target substrate also improves adhesion and mechanical robustness under bending and handling, which is critical for flexible and wearable device applications.

iGii's process is a low-energy electrically powered process, foregoing the use of mined metals and toxic chemicals. It utilises 0.026 kWh per cm^2^ of the 3DG film, which can be as low as 9.3 kWh kg^−1^ compared to CVD, which can be 80.3 kWh kg^−1^.^46^ The process can be powered using 100% renewable energy wherever available, and the environmental impact of the 3DG has been carefully researched. In comparison to other methods of generating graphene-related materials such as CVD and mechanical exfoliation, iGii's process grows 3DG directly onto the target substrate, removing the need for additional steps such as delamination from the substrate for CVD graphene and additional formulation of mechanically or chemically exfoliated graphene into inks for printing onto the substrate of choice. Not only does 3DG require less energy to produce but it also takes away the need for additional processing which also requires energy and resource.

## 4. Conclusion

In this work, two structurally distinct three-dimensional graphene foams with high and low electrical resistivity were systematically investigated to elucidate the interplay between network architecture, chemical composition, optical response, and electrochemical performance. By combining electron microscopy, AFM, Raman spectroscopy with multiple excitation wavelengths, surface chemical analysis, broadband optical characterization, and electrochemical testing, a comprehensive structure–property–function relationship has been established for these complex 3D carbon networks.

Morphological analysis revealed that the high defect graphene foam (HDGF) possesses a more open, heterogeneous, and highly porous architecture, having a surface roughness of 18 nm, whereas the low defect graphene foam (LDGF) exhibits a denser, more uniform, and better-connected nanosheet network, having a surface roughness of 4.5 nm. This aligns with the observations from the SEM images.

Chemical and surface analyses confirmed that both materials are predominantly carbon-based, with differences in oxygen content and sp^2^/sp^3^ bonding ratios. The higher sp^2^ carbon fraction and reduced oxygen content in LDGF correlate well with its enhanced electrical conductivity, while the greater degree of functionalization and porosity in HDGF contributes to its higher electrochemical capacitance. Wettability measurements further highlighted these contrasts, with HDGF exhibiting super hydrophilic behavior, with a contact angle of 14°, facilitating rapid electrolyte access, while LDGF remained moderately hydrophilic at 72°.

A key outcome of this study is the discovery of a pronounced wavelength-dependent Raman response in graphene foams. Multi-wavelength Raman spectroscopy revealed an unusual “optical switch” behavior, where the *I*_D_/*I*_G_ intensity ratio changes sharply with excitation energy. This effect is attributed to excitation-dependent resonance of defect-related phonon modes and differences in local disorder and electronic structure within the 3D network. Such behavior has not been previously reported for bulk graphene foams and highlights the importance of excitation wavelength when interpreting Raman metrics in highly disordered, three-dimensional graphene systems.

Broadband optical measurements demonstrated that both graphene foams exhibit near-zero transmittance and reflectance from the visible through the long-wave infrared region, with high defect GF showing a lower average reflectance (0.028%) over the visible region as compared with the low defect GF (0.153%), indicating exceptionally strong light absorption and trapping across a wide spectral range. These properties, combined with the mechanically flexible Kapton substrate, position both materials as highly attractive candidates for broadband optical absorbers and stray-light suppression coatings in optical and photonic systems.

Electrochemical characterization showed that both foams behave as ideal electric double-layer capacitors with excellent rate capability and low series resistance. While LDGF benefits from lower internal resistance and superior rate retention, HDGF delivers a higher areal capacitance of 38 µF cm^−2^ due to its more developed porous structure, illustrating a clear trade-off between conductivity and charge storage capacity governed by network morphology.

Overall, this study demonstrates that controlled variations in the architecture and chemistry of 3D graphene foams enable fine-tuning of their electrical, optical, and electrochemical properties. The identification of excitation-dependent Raman “optical switching,” coupled with broadband optical absorption and robust electrochemical performance, sets a new benchmark for the systematic characterization of graphene foams. These findings not only deepen fundamental understanding of transport and light–matter interactions in 3D graphene networks but also highlight their strong potential for applications in energy storage, optical modulators, photonic devices, sensing platforms, and stray-light management technologies.

## Author contributions

Emma Keel: conceptualization, methodology, validation, investigation, Raman spectroscopy measurement and analysis, electrochemical measurements, formal analysis, and writing (original draft preparation, review and editing); Michal Mazur: XPS and SEM measurements and analysis; Jarosław Domaradzki: *I*–*V* and wettability measurements and analysis; Damian Wojcieszak: AFM and EDS measurements and analysis; Lewis Fleming: optical property measurements and analysis; Qaisar Abbas: supervision, electrochemical measurements and analysis; Michelle Ntola: original draft preparation, writing, review and editing; Marco Caffio: supervision, project administration. Carlos García Núnez: supervision, draft preparation, review and editing. Des Gibson: supervision, draft preparation, project administration, writing, review and editing, corresponding author.

## Conflicts of interest

There are no conflicts to declare.

## Data Availability

The data that support the findings of this study are available from the corresponding author, upon reasonable request.
